# Tritium-Labeled Compounds. XI. Mechanism for the Oxidation of Aldehydes and Aldoses-*1*-*t* With Sodium Chlorite^1^

**DOI:** 10.6028/jres.068A.027

**Published:** 1964-06-01

**Authors:** Horace S. Isbell, Lorna T. Sniegoski

## Abstract

Measurements of kinetic isotope-effects (k*/k) for the oxidation of ten aldoses-*1-t* with sodium chlorite in acid solution gave values ranging from 0.56 to 0.75, with an average of 0.66. The values show that the C1—H* bond is not ruptured in the rate-determining step. For the reaction, a mechanism is proposed which accounts for this fact and for the dependence of the rate of reaction on the concentration of chlorous acid. The mechanism involves formation of a chlorous acid—aldehyde intermediate; this decomposes, giving the aldonic acid and hypochlorous acid. The latter then reacts with more chlorous acid, affording the chlorine dioxide and hydrogen chloride found experimentally.

## 1. Introduction

Sodium chlorite is widely used for the technological treatment of carbohydrates [1],^2^ but the basic chemistry of the reactions involved has not been adequately developed. Jeanes and Isbell [2] showed that (a) nonreducing saccharides, alditols, ketoses, and aldonic acids are inert to chlorites in acid solution, whereas aldoses are oxidized to aldonic acids; (b) the first detectable product from the aldose is the free aldonic acid, and not the lactone; (c) aldopentoses are oxidized more rapidly than aldohexoses, and monosaccharides more rapidly than disaccharides; and (d) the anomer of d-glucopyranose that, in its most stable conformation, is now known to have an axial C1-hydroxyl group is oxidized more rapidly than the anomer having an equatorial C1-hydroxyl group. Formation of the free acid as an initial product, and the more-rapid oxidation of the anomer having an axial C1-hydroxyl group, indicate that the reaction does *not* involve the direct oxidation of a pyranose intermediate, in contrast to the oxidation of an aldose with bromine [3].

Laurier, Wilson, and Flynn [4] established that the oxidation is first-order with respect to both aldose and chlorous acid, and they developed a procedure for the quantitative determination of aldoses from the amount of chlorine dioxide formed in the reaction. For d-glucose, d-galactose, and d-mannose, Wilson [5] found an approximate parallelism between the rates of oxidation and the rates of transformation of the ring form of the aldose to the aldehyde form. Previously, White, Taylor, and Vincent [1] had reported that sodium chlorite in acid solution rapidly oxidizes formaldehyde, acetaldehyde, and benzaldehyde, with the formation of chlorine dioxide. Thus, it seems likely that the oxidation of aldoses takes place through the aldehyde form. The situation is complicated by concurrent decomposition of chlorous acid and by certain disproportionation reactions involving chlorine dioxide, chlorous acid, and chlorates. After making allowance for these complications, Jeanes and Isbell [2] found that the oxidation follows eq (1).

$$\mathrm{RCHO}+3\;{\mathrm{HClO}}_{2}\to {\mathrm{RCO}}_{2}H+\mathrm{HCl}+2\;{\mathrm{ClO}}_{2}+H_{2}O.$$ (1)

A satisfactory mechanism for the oxidation of the aldose (or aldehyde) must account for the utilization of 3 moles of chlorous acid per mole, in a process that is first-order with respect to chlorous acid and the aldehyde. In the reaction of an aldehyde with a chlorite in acid solution, one possibility would be a nucleophilic addition of the chlorite ion to the conjugate acid as shown in eq (2). Formation of this

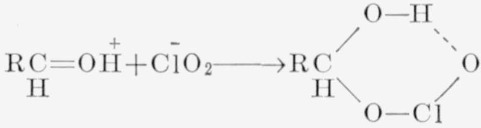
(2)intermediate would account for the fact that the reaction is first-order with respect to chlorous acid and aldehyde. The intermediate would be similar to an aldehyde bisulfite compound and, by hydrogen bonding, could have a cyclic structure. The intermediate would be unstable, because of the electron deficiency of the chlorine atom and the presence of a potential source of electrons at the C1—hydrogen bond. Release of the C1—hydrogen atom, with the redistribution of electrons as indicated in eq (3), would give a carboxylic acid and hypochlorous acid.

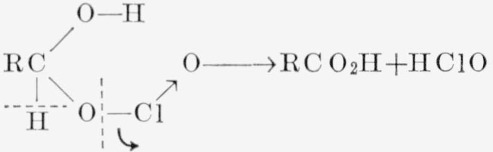
(3)One mole of the nascent hypochlorous acid would then react with two moles of chlorous acid in the known manner, according to eq (4).

$$\mathrm{HClO}+2\;{\mathrm{HClO}}_{2}\to 2\;{\mathrm{ClO}}_{2}+\mathrm{HCl}+H_{2}O$$ (4)

The overall process (eq (3) plus eq (4)) corresponds to eq (1).

With aldoses, the reaction includes the following steps: (1) formation of the aldehyde intermediate from the pyranose; (2) combination of chlorite ion with the aldehyde cation; (3) decomposition of the aldehyde—chlorite complex, yielding aldonic acid and hypochlorous acid; and (4) reaction of 1 mole of the resulting hypochlorous acid with 2 moles of chlorous acid, affording 2 moles of chlorine dioxide and 1 mole of hydrogen chloride. Side reactions give small proportions of chlorate and oxalic acid, as noted and explained by Jeanes and Isbell [2].

Formation of a chlorous acid—aldose intermediate accounts for the first-order kinetics found by Launer, Wilson, and Flynn [4], but, from the kinetics, it is not possible to predict which step is rate determining. The problem can be attacked, however, by the use of isotope effects in the oxidation of aldoses-*1-t.*

In the oxidation of aldoses-*1*-*t* by bromine and iodine, we have shown [6, 7] that the isotope effect is about 0.14 for reactions in which cleavage of the C1—hydrogen bond is rate determining, and about 0.8 for reactions in which isomerization is rate determining. Hence, an isotope effect of about 0.8 would be expected if steps 1 or 2 are rate determining and of about 0.14 if step 3 is rate determining.

Results for the oxidation of ten aldoses-*1*-*t* with sodium chlorite, in slightly acid solution at 0 °C, are given in table 1. The values 3 of k*/*k* average 0.66.

The magnitude of the isotope effect clearly shows that the C1—hydrogen bond is not broken in the rate-determining step. Presumably the aldehyde— chlorous acid complex forms slowly (steps 1 and 2) and collapses rapidly (step 3). The rate of formation of the aldehyde intermediate from the pyranose by the mutarotation reaction (step 1) depends on the presence of acid and base catalysts and on the particular aldose involved. Step 2 is sensitive to *p*H and to the concentration of chlorite. Hence, the relative rates for steps 1 and 2 would be expected to depend on the experimental conditions and on the particular aldose involved.

The difference in the rates of reaction for the *α* and *β* anomers of d-glucopyranose, reported by Jeanes and Isbell [2], shows that reaction of the intermediate aldehyde with chlorous acid is sufficiently fast to preclude maintenance of a steady state in which the aldehyde is in equilibrium with all modifications of the sugar. According to the transition-state theory [8], the rate of reaction depends on the difference in free energy (Δ*F*‡) for the reactants in the ground state and in the transition state. The higher rate of reaction for *α*-d-glucopyranose indicates that the difference in free energy is less for the reaction of *α*-d-glucopyranose than for the reaction of *β*-d-glucopyranose. This might be expected, because, in the ground state, *β*-d-glucopyranose is at a lower energy level than *α*-d-glucopyranose, and the difference in the transition states is small.

At present, there are few data for the chlorite oxidation of the anomers of the other aldoses. For different aldoses, there is an approximate parallelism between the rates of oxidation and the rates of isomerization [5]. Quantitative correlations are not feasible, however, because the reactions are complex and because, in most instances, the isomerization and oxidation reactions have not been measured under like conditions.

## 2. Experimental Procedures

### 2.1. Materials and Apparatus

#### a. Labeled Aldoses

Aldoses-*1*-*t* were prepared by the procedures given in reference [9]. d-Glucose-*6*-*t* and d-glucose-*6*-*C*^14^ were prepared by the procedures given in references [10] and [11], respectively. The “doubly labeled” aldoses d-glucose-*1*-*C*^14^-*6*-*t* and d-glucose-*1*-*t*-*6*-*C*^14^ were prepared by mixing suitable quantities of the singly labeled sugars. The mixtures were recrystallized from water with the addition of methanol and 2- propanol. Before use of each sugar, the purity was checked by paper chromatography.

#### b. Chlorous Acid Solution

A *1-M* solution of sodium chlorite was prepared by dissolving 9.5 g of commercial sodium chlorite in 100 ml of water. The solution of chlorous acid for the oxidation was prepared immediately before use by mixing one volume of ice-cold, *1-M* sodium chlorite with 1.1 volumes of *1-M* acetic acid; the freshly prepared solution had a *p*H of 3.4.

#### c. Counting Equipment

Carbon 14 or tritium, or both, were determined by measurements of radioactivity, either with a liquid scintillation spectrometer or with a windowless, gas-flow, proportional counter. The measurements given in table 1 were made with the liquid scintillation spectrometer by the procedure given in reference [12]. Those given in table 2 were made, in films, with the gas-flow, proportional counter by the procedure described in [13], and with the liquid scintillation spectrometer by the procedure described in [6] and [12].

### 2.2. Chlorous Acid Oxidation of Aldoses-*1*-*t*

#### a. Determination of k*/*k* by the H_2_O-*t* Method

A 0.05-mmole sample of aldosc-*1-t* was weighed into a 10-ml volumetric flask. The aldose-*1-t* was dissolved in three drops of water, and the solution was allowed to stand overnight. The flask was then cooled in an ice bath, and 1.58 ml (a fivefold excess) of ice-cold, chlorous acid solution was added. The reaction was allowed to proceed for sufficient time to oxidize 20 to 45 percent of the aldose-*1*-*t*, the solution was diluted to 10 ml with ice water, and aliquots were taken for analysis. A 5-ml aliquot was transferred to a 25-ml volumetric flask containing 0.83 ml of 2.11-*N* potassium iodide. A 2-ml aliquot was removed to a 50-ml standard-tapered, male-joint flask containing 20 mg of anhydrous sodium carbonate. This solution was frozen and the water-*t* was sublimed by the procedure described in [6]. Duplicate samples of the tritiated water were analyzed with the Packard Tri-Carb counter, using 100 *μ*l of distillate and 10 ml of the scintillator solution. Each sample was counted to a total of 10,000 counts, to give a statistical accuracy of ±1 percent.

The 5-ml aliquot in the 25-ml flask was used for the determination of the residual aldose-*1*-*t*. The iodine formed by reaction of the excess chlorous acid with the potassium iodide was reduced by the addition of 6 to 8 ml of a 6-percent, aqueous solution of sulfur dioxide. The solution was allowed to stand for at least half an hour, and was then boiled to remove all sulfur dioxide and to concentrate the solution. After the solution had cooled, it was neutralized with aqueous sodium hydroxide to the Phenol Red endpoint. The volume was adjusted to 25 ml, and three 5-ml aliquots were analyzed for reducing sugar by the Somogyi method [14]. The method was standardized, for each aldose, and the factor so obtained was used for calculating the amount of aldose in the sample.

The isotope effect, k*/*k*, was calculated from eq (5),

k*/k=log(1−rf)/log(1−f) (5)

where *f* is the ratio of the weight of aldose consumed to the initial weight, and *rf* is the ratio of the total *μ*c of tritiated water formed to the *μ*c of tritium in the initial sample (calculated from the specific activity of the sample).

#### b. Determination of k*/*k* by the Double-Labeled Method [15]

Samples of doubly labeled d-glucose of about 9 mg (0.05 mmole) were weighed into separate, small, test tubes on the microbalance. The tubes were placed on a shaker in an ice-water bath, and the quantities of ice-cold chlorous acid solution listed in table 2 were added. The reactions were allowed to proceed for the times given.

After elapse of the recorded reaction time, 100 mg of d-glucose carrier and 50 mg of a decolorizing carbon were added to each tube, the mixture was filtered, and the filtrate was passed through mixed anioncation resin. The eluate and washings were concentrated, and filtered into a standard-tapered test tube. The solution was concentrated and the labeled d-glucose was crystallized three times from water, with the addition of methanol and 2-propanol. The purified, doubly labeled d-glucose was assayed for radioactivity, either in solution with the liquid scintillation counter, or in films with the proportional counter.

The isotope effect, k*/*k*, was calculated by eq (6) [15],

k*/k=1+[log(p/p°)/log(1−f)] (6)

where *p* is the ratio of the functional to the reference isotope in the residual reactant, *p°* is the ratio in the initial reactant, and *f* and k*/*k* have the same meanings as before. The data are recorded in table 2.

## Figures and Tables

**Table 1:** Isotope effects for the oxidation of aldoses-*1*-*t* with sodium chlorite at *pH* 3.4 and 0 °C

Aldos*e-1-t*	Reaction time	Extent of oxidation (*f*)	Isotope effect, k*/*k*	Average
				
	*min*			
l-Arabinose-*1*-*t*	10	0.279	0.69	
	15	.254	.73	0. 71
d-Galactose-*1*-*t*	60	.302	.56	
	70	.302	.67	
	80	.297	.76	.66
d-Glucose-*1*-*t*	180	.243	.64	
	240	.285	.70	.67
d-Lyxose-*1*-*t*	7	.248	.66	
	10	.463	.71	.68
d-Mannose-*1*-*t*	45	.399	.59	
	60	.446	.57	.58
l-Rhamnose-*1*-*t* · H_2_O	30	.273	.53	
	60	.342	.61	
	90	.447	.67	.60
d-Ribose-*1*-*t*	3	.209	.70	
	5	.338	.73	.72
d-Talose-*1-t*	10	.330	.73	
	15	.394	.77	.75
d-*X*ylose-*1*-*t*	20	.281	.52	
	25	.263	.60	.56
Average				0.66

**Table 2:** Isotope effects for the oxidation of d-glucose-*1*-*C*^14^-*6*-*t* and of d-glucose-*1*-*t*-*6*-*C*^14^ with sodium chlorite at *pH* 3.4 and 0 °C

Labeled aldose	Sample		HClO_2_	Reaction time	Carrier added	*f*	*p°*	*p*	k*/*k*	k*/*k* (average)
										
	*mg*	*m mole*	*m mole*	*hr*	*mg*					
d-Glucose-*1*-*C*^14^-*6*-*t*	11.370	0.063	0.170	20	100.2	0.752	0.1144	0.1105	1.02	
	9.481	.053	.120	20	100.4	.656	.1144	.1164	0.98	1.00
d-Glucose-*1-t-6-C*^14^	9.825	.055	.147	20	100.1	.764	9.12	14.40	.68	
	9.155	.051	.120	20	100.4	.695	9.12	13.84	.65	
	a 9.043	.050	.151	1	99.9	.306	9.12	10.00	.75	
	9.190	.052	.294	5.5	100.1	.437	9.12	11.12	.66	
	9.807	.054	.147	20	100.8	.680	8.02	11.12	.71	
	9.545	.053	.127	20	100.8	.640	8.02	10.78	.71	
	11.030	.061	.918	8.5	100.6	.611	8.02	9.88	.78	
	10.217	.057	.851	7.5	100.8	.596	8.02	10.10	.75	0.72

a Phosphoric acid was used in the reaction mixture, instead of acetic acid.

## Footnotes

2 Figures in brackets indicate the literature references at the end of this paper.

3 The variation in the values (0.56 to 0.75) found for different aldoses-*1-t* is not significant, because it does not differ widely from that found in replicate measurements with a single aldose-*1*-*t* (for example, 0.56 to 0.76 for d-galactose-*1*-*t*). The relatively large variation arises from the difficulty in determining small quantities of reducing sugar in mixtures containing sodium chlorite.
